# Determining the Impact of Nonionizing Electromagnetic Waves on Human Pregnancy and Teratogenicity: Protocol for Systematic Review and Meta-Analysis

**DOI:** 10.2196/86479

**Published:** 2026-03-30

**Authors:** Desy Armalina, Neni Susilaningsih, Heri Sutanto, Sunarno Sunarno

**Affiliations:** 1 Doctoral Study Program of Medical and Health Science, Universitas Diponegoro, Jl. Prof. Soedarto, Tembalang, Kec. Tembalang, Kota Semarang, Jawa Tengah, Semarang, Central Java, 50275, Indonesia, 62 08112741612, 62 2476928010; 2 Anatomy-Histology Department, Faculty of Medicine, Universitas Diponegoro, Semarang, Indonesia; 3 Department of Physics, Faculty of Science and Mathematics, Universitas Diponegoro, Semarang, Indonesia; 4 Department of Biology, Faculty of Science and Mathematics, Universitas Diponegoro, Semarang, Indonesia

**Keywords:** electromagnetic waves, nonionizing radiation, pregnancy, teratogenicity, systematic review, mobile phone

## Abstract

**Background:**

The widespread use of mobile devices has markedly increased global exposure to nonionizing electromagnetic waves (EMWs). Emerging evidence indicates potential biological effects of EMW exposure in susceptible populations, particularly pregnant women; however, findings remain inconsistent.

**Objective:**

This protocol delineates a systematic review aimed at synthesizing and critically evaluating the teratogenic and pregnancy-related effects of nonionizing EMW exposure in pregnant women.

**Methods:**

This protocol adheres to the PRISMA-P (Preferred Reporting Items for Systematic Review and Meta-Analysis Protocols) 2020 guidelines and has been registered with the International Prospective Register of Systematic Reviews (PROSPERO; CRD42023475665). A comprehensive literature search will be conducted in PubMed/MEDLINE, Scopus, Web of Science, Embase, ScienceDirect, SpringerLink, Wiley Online Library, and Google Scholar, with supplementary searches of the World Health Organization International Clinical Trials Registry Platform and ClinicalTrials.gov. Eligible studies will include pregnant women exposed to nonionizing EMWs from mobile phones and related wireless devices. The primary outcomes will be pregnancy complications and fetal anomalies, with secondary outcomes assessed as previously reported. Study selection, data extraction, and risk of bias assessment will be performed independently by 2 reviewers. Where appropriate, a random-effects meta-analysis will be conducted.

**Results:**

Funding for this study was secured in March 2026. The literature search and study screening are planned for April to July 2026, with data extraction, risk of bias assessment, and synthesis expected to be completed by September 2026. The final results are anticipated to be submitted for publication in late 2026.

**Conclusions:**

This systematic review is expected to provide consolidated evidence on the potential teratogenic and pregnancy-related effects of nonionizing EMW exposure, thereby supporting future research and evidence-based recommendations for public health.

## Introduction

Global digital transformation has fundamentally altered human interactions with electromagnetic energy. Modern lifestyles are now inseparable from mobile phones, Wi-Fi routers, base stations, and wireless devices, all of which emit nonionizing electromagnetic waves (EMWs) in the radiofrequency (RF) and microwave spectra. The World Health Organization has noted that electromagnetic fields (EMFs) are among the most common and fastest-growing environmental exposures worldwide, with population-level exposure continuing to increase as wireless technologies expand. Although nonionizing radiation lacks the quantum energy necessary to break chemical bonds or ionize atoms, experimental and dosimetric studies have suggested that exposure may induce biological effects through thermal mechanisms related to tissue heating and nonthermal interactions observed under specific exposure conditions, including oxidative stress responses and alterations in cellular signaling pathways [1,2].

Nonionizing EMWs are typically defined as waves with frequencies ranging from 300 Hz to 300 GHz. At these frequencies, the absorbed energy can result in localized tissue heating, which represents the most well-characterized biological effect of EMW exposure. Nonthermal biological interactions, including perturbations in redox balance, cellular signaling, and gene expression, have also been reported in experimental models; however, these findings remain inconsistent, and their relevance to human pregnancy outcomes has not been conclusively established [1,2].

Mobile phones, as ubiquitous sources of EMW exposure, have become integral to our daily life. Global smartphone use has increased from a few hundred million users in 2008 to more than 6.5 billion active users by 2023. In parallel, the average daily usage time has increased substantially, particularly during and after the COVID-19 pandemic [3].

Recent epidemiological studies have explored the potential associations between maternal mobile phone use and adverse pregnancy outcomes, including miscarriage, preterm birth, and abnormal birth weight; however, the results remain heterogeneous across populations and study methodologies [4].

Consequently, the intensity, proximity, and duration of EMW exposure in the general population, particularly among pregnant women, has increased over time. Pregnancy represents a critical window of biological vulnerability due to the complex endocrine, immunological, and vascular adaptations required to sustain fetal growth. The developing embryo and fetus may be especially sensitive to environmental exposures because of rapid cellular proliferation, immature antioxidant defenses, and limited detoxification capacity. As a result, EMW exposure during pregnancy has been investigated for its potential effects on placental function, uteroplacental blood flow, embryonic development, and fetal morphogenesis [5-7].

Importantly, the presence of a potential biological mechanism does not imply a clinically relevant risk. The current international consensus indicates that adverse health effects from nonionizing EMW exposure are strongly dependent on exposure intensity, duration, and proximity to the source. At high power densities, such as in occupational or accidental settings near strong RF emitters, thermal tissue injury may occur; however, under typical environmental and consumer-use conditions, exposure levels are substantially lower and generally remain below established safety limits [8,9]. Consequently, while EMWs represent a potential hazard under specific conditions, the actual health risk during normal daily use is determined by the dose and duration of exposure, which are typically far below the thresholds known to cause harm. This distinction between hazard and risk is central to the interpretation of existing evidence.

Epidemiological investigations have reported mixed findings regarding the association between EMF exposure and adverse pregnancy outcomes. A meta-analysis by Ghazanfarpour et al [3] reported an increased risk of miscarriage associated with higher levels of extremely low-frequency electromagnetic exposure. Similarly, Li et al [10] observed an association between magnetic field exposure and spontaneous abortion in a prospective cohort study. In addition, computational dosimetric modeling by Takei et al [11] demonstrated that mobile phone radiation may increase localized placental and fetal temperatures under specific exposure scenarios, suggesting a potential thermal pathway warranting further investigation.

In contrast, other large-scale epidemiological studies have not identified significant associations between EMW exposure and adverse pregnancy or birth outcomes, highlighting substantial heterogeneity in exposure assessment, study design, and confounder control [5,12]. Many earlier studies were also limited by small sample sizes, indirect exposure metrics, and incomplete adjustment for potential confounders, including maternal stress and environmental coexposure.

From a mechanistic perspective, experimental studies have reported that EMW exposure may induce oxidative stress responses, alter antioxidant enzyme activity, and influence calcium signaling and apoptotic pathways in animal and in vitro models [13-16]. However, the extent to which these experimental findings translate to clinically meaningful effects during human pregnancy remains unclear.

Given the complex and often contradictory findings in the existing literature, a rigorous systematic review is necessary. This systematic review protocol aims to systematically synthesize and critically evaluate the available evidence on the teratogenic and pregnancy-related effects of nonionizing EMW exposure, particularly from mobile phone use, in pregnant women.

## Methods

### Protocol and Registration

This review adheres to the PRISMA-P (Preferred Reporting Items for Systematic Review and Meta-Analysis Protocols) 2020 standards [17] and is registered with International Prospective Register of Systematic Reviews (PROSPERO; ID: CRD42023475665), which ensures transparency and reproducibility. The completed PRISMA 2020 checklist is provided in Checklist 1. All protocol amendments will be documented in PROSPERO.

### Eligibility Criteria

Studies will be selected according to the Population Intervention (or Exposure, for observational studies) Comparator Outcomes Study design (PICOS) framework as follows.

#### Population

Included were pregnant women (any maternal age) and their offspring.

Outcome time window: Outcomes will be eligible if measured during pregnancy (in utero) and/or at birth (perinatal/neonatal periods). If studies report outcomes beyond birth, outcomes will be included up to 28 days postpartum (neonatal period) and analyzed separately as postnatal outcomes. Outcomes measured beyond 28 days will not be included in the primary synthesis but may be described narratively if directly relevant.

#### Intervention: Exposure (EMW/EMF)

The included sources were nonionizing EMW exposure related to mobile phone use and related consumer wireless devices (eg, smartphones and Wi-Fi–enabled devices) and/or ambient RF sources, where exposure was quantified or reasonably categorized.

Exposure metrics: Studies will be eligible if exposure is reported using at least one of the following: specific absorption rate (SAR), power density (W/m² or mW/cm²), field strength (V/m), magnetic flux density (µT/mG), or a validated exposure classification method (eg, job-exposure matrix or personal dosimetry).

Dose range limits: We will not impose a single universal lower or upper cutoff for power density a priori because consumer and environmental studies use heterogeneous exposure metrics and thresholds. Instead, we will extract the exposure intensity and categorize it into low or typical consumer use versus higher occupational or accidental exposure levels as defined by each study, and we will interpret the findings in relation to established safety guidance (eg, ICNIRP/WHO) in the Discussion. Where data permit, dose–response analyses will be conducted according to the exposure strata.

Comparators: Lower exposure group or nonexposed group, depending on study design.

#### Outcomes

The primary outcomes of this review will be preterm birth and major congenital anomalies, assessed at birth. These outcomes were selected a priori based on their clinical relevance and biological plausibility in relation to nonionizing EMW exposure. Secondary outcomes will include miscarriage, stillbirth, low birth weight, small for gestational age, placental outcomes (as reported), and neonatal outcomes up to 28 days postpartum. Outcomes reported beyond the neonatal period will be described narratively when relevant but will not be included in the primary quantitative synthesis.

#### Timing

Timing will be a consideration.

- Minimum exposure or observation duration: Studies must report exposure during pregnancy with an identifiable exposure window (eg, trimester-specific or pregnancy-level exposure).
- Timing categories: We will extract and categorize exposure timing by trimester (if available) and duration of exposure (eg, daily use duration or cumulative exposure).
- Follow-up window: In utero, at birth, and up to 28 days after birth.

#### Study Design

Included: Human primary research only—randomized trials (if any), cohort studies, case-control studies, cross-sectional studies, and case-crossover designs that reported pregnancy outcomes.

Excluded from quantitative synthesis (meta-analysis): Systematic reviews, narrative reviews, editorials, letters, case reports, and all animal or in vivo studies.

Use of systematic reviews: Systematic reviews will be used only for citation tracking to identify additional eligible primary human studies but will not be included as studies in the meta-analysis.

### Comparator

The comparator groups will include pregnant women with no reported exposure to nonionizing EMWs, background environmental exposure only, or the lowest exposure category defined within each individual study. The comparator definitions will be extracted as reported and assessed for comparability across studies.

### Exclusion Criteria

#### Insufficient Exposure Information

Studies will be excluded if EMW exposure is not defined in sufficient detail to allow meaningful interpretation or comparison. Specifically, studies will be considered to have insufficient exposure data if none of the following are reported or inferred:

- Exposure frequency band (eg, extremely low-frequency, RF, microwave, or device-specific frequency range)
- Timing or duration of exposure during pregnancy (eg, trimester-specific exposure, daily use duration, cumulative exposure, or occupational exposure period)

Studies that report qualitative or proxy exposure measures (eg, categorized phone use and job-exposure matrices) will not be excluded solely on this basis, provided that the exposure classification is clearly described and consistently applied.

#### Language

No language restrictions were applied during the screening stage. Studies published in any language will be considered. Non-English studies will be screened using English abstracts where available, and, when necessary, machine-assisted translation (eg, Google Translate or equivalent tools) will be used. For studies deemed potentially eligible after full-text screening, translations will be used to extract relevant data, with attention to methodological clarity and outcome definition.

### Search Strategy and Information Sources

The search strategy will be developed and refined in consultation with an experienced university health sciences librarian to ensure methodological rigor and comprehensive coverage of the literature. The final search strategy will combine controlled vocabulary (eg, Medical Subject Headings terms) and free-text keywords related to EMWs, pregnancy, and reproductive outcomes and will be adapted for each database.

The electronic databases to be searched include PubMed/MEDLINE, Scopus, Web of Science, Embase, ScienceDirect, SpringerLink, Wiley Online Library, and Google Scholar.

In addition, clinical trial registries will be searched, including the WHO International Clinical Trials Registry Platform. Where relevant, other registries, such as ClinicalTrials.gov, will be screened to identify completed or ongoing studies with unpublished results.

In addition to bibliographic databases, gray literature sources will be searched to minimize publication bias. These include ClinicalTrials.gov and the WHO International Clinical Trials Registry Platform. Embase and the Cochrane Library are included to ensure comprehensive coverage of biomedical, epidemiological, and clinical trial literature not fully indexed in PubMed.

### Study Selection and Data Extraction

Two reviewers will independently assess titles and abstracts for relevance. Full texts will then be reviewed for eligibility. Discrepancies will be resolved through discussion or by a third reviewer.

**Figure 1.**
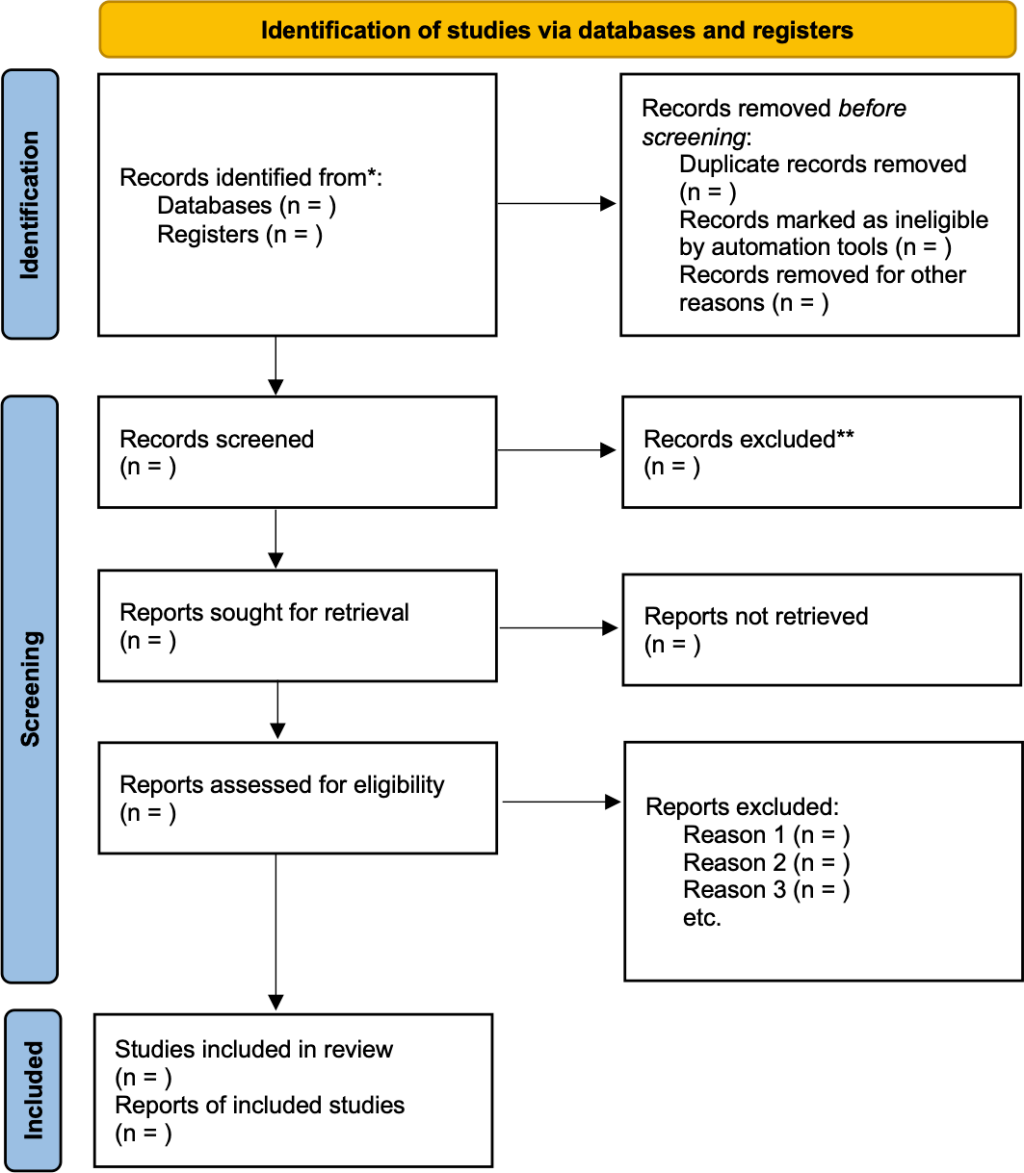
PRISMA (Preferred Reporting Items for Systematic Review and Meta-Analysis) 2020 flow diagram for new systematic reviews, which included searches of databases and registers only. The asterisk (*) refers to records identified through database searching (PubMed/MEDLINE, Scopus, Web of Science, Embase, ScienceDirect, SpringerLink, Wiley Online Library, and Google Scholar). The double asterisk (**) refers to records excluded during title/abstract screening.

The selection process will be documented using a PRISMA 2020 flowchart (Figure 1).

Data will be extracted into Microsoft Excel, capturing the following:

1. Study characteristics (author, year, country)
2. Study design and sample size
3. Exposure parameters (frequency, intensity, duration)
4. Participant details (age, gestational week)
5. Outcomes and key findings
6. Reported statistical measures (odds ratio, risk ratio, CI)

When information is incomplete, the authors will be contacted for clarification.

### Risk of Bias and Quality Assessment

Only primary human studies will be included in the risk of bias assessment and quantitative synthesis. The risk of bias in nonrandomized observational studies will be assessed using the Risk Of Bias In Nonrandomized Studies of Interventions tool, which evaluates bias across the domains of confounding, participant selection, exposure classification, deviations from intended exposure, missing data, outcome measurement, and selective reporting. Where randomized controlled trials are identified, the Cochrane Risk of Bias 2 tool will be applied. Each study will be categorized as having low, moderate, serious, or critical risk of bias according to the tool’s guidance. Studies judged to be at critical risk of bias will be excluded from quantitative synthesis but described narratively. Reporting quality will be evaluated descriptively using the Strengthening the Reporting of Observational Studies in Epidemiology (STROBE) checklist, without influencing risk of bias judgments.

### Data Synthesis and Assessment of Heterogeneity

Statistical heterogeneity will be assessed using the *I*² statistic and chi-square test. In accordance with the Cochrane Handbook, *I*² values will be interpreted cautiously and in context, recognizing that fixed thresholds can be misleading and that the importance of heterogeneity depends on multiple factors, including the magnitude and direction of effects and the strength of evidence for heterogeneity (eg, CIs around *I*² and the number of included studies).

Meta-analyses will be conducted using random-effects models when appropriate. Rather than relying on rigid *I*² cutoffs to define “substantial” heterogeneity, heterogeneity will be primarily addressed through clinical and methodological assessments, including differences in study populations, exposure metrics, outcome definitions, and confounder adjustment.

To better reflect the between-study variability and the expected range of true effects across different settings, prediction intervals will be calculated and reported for random-effects meta-analyses, in line with contemporary methodological guidance [18].

### Statistical Analysis

When quantitative synthesis is feasible, a random-effects meta-analysis will be performed using the DerSimonian–Laird or restricted maximum likelihood approach, depending on the data structure. Effect estimates will be reported with 95% CIs and, where applicable, with prediction intervals. Sensitivity analyses will be conducted to assess the robustness of the findings to study quality and exposure definitions.

### Observational Data and Minimum Requirements for Confounding Control

Given that the available evidence is predominantly observational, included studies must demonstrate a priori adjustment for key confounders to be eligible for quantitative synthesis. At a minimum, studies must adjust for the following:

- maternal age
- gestational age
- socioeconomic status or education
- smoking and/or alcohol use
- at least one indicator of overall health or pregnancy risk (eg, parity or comorbidities)

Studies failing to account for these minimum confounders will be excluded from meta-analysis and described narratively only. This minimum adjustment set will be applied a priori as a criterion for inclusion in quantitative synthesis. Residual confounding will be explicitly considered in the interpretation of findings, and causal language will be avoided in accordance with the limitations of observational evidence.

### Certainty of Evidence

The overall quality and certainty of evidence for each outcome will be graded using the GRADE (Grading of Recommendations Assessment, Development, and Evaluation) system as high, moderate, low, or very low [19].

### Ethical Considerations

This study is a systematic review protocol based exclusively on the published literature and does not involve the collection of primary data or interaction with human participants. Therefore, ethical approval from an institutional review board or ethics committee is not required, in accordance with institutional and international guidelines for research involving publicly available data.

## Results

This protocol was registered with PROSPERO in 2023 and finalized in March 2026 following funding approval. The literature search and study selection are planned for April to July 2026, followed by data extraction, risk of bias assessment, and data synthesis by September 2026. Submission of the final systematic review manuscript is anticipated in late 2026.

## Discussion

### Expected Findings

This systematic review protocol provides a transparent methodological framework for evaluating the potential effects of nonionizing EMWs on human pregnancy and fetal development. Given the rapid expansion of mobile phone use and wireless technology, understanding the biological implications of prolonged exposure to EMWs has become an increasingly important public health concern [1,2]. Despite extensive research, the existing literature remains heterogeneous, with findings ranging from no observable adverse effects to possible reproductive or developmental impacts [13,17,20]. This protocol is designed to systematically assess and contextualize these inconsistencies through a rigorous methodological appraisal.

### Comparison With Prior Work

Biological tissues can absorb EMWs, with thermal effects related to localized tissue heating representing the most well-characterized interaction [21]. In addition, nonthermal biological interactions have been described primarily in experimental and in vitro models, including oxidative stress responses, calcium channel modulation, and alterations in gene expression; however, these findings remain inconsistent, and their relevance to human pregnancy outcomes is not firmly established [1,2].

Previous human studies have reported mixed associations between electromagnetic exposure and adverse pregnancy outcomes, including miscarriage and fetal growth restriction [3,12]. A systematic review by Wdowiak et al [14] reported potential links between chronic EMF exposure and lower birth weight, placental dysfunction, and developmental delay. Similarly, computational modeling by Takei et al [11] suggested that smartphone radiation may increase localized placental temperature, potentially affecting placental perfusion. Advanced modeling studies have further explored how RF radiation from commonly used mobile phones may influence electromagnetic exposure profiles in pregnant women, highlighting potential heat distribution effects that could have biological relevance; however, these findings require empirical validation in human populations [22].

Conversely, other large-scale epidemiological studies have not confirmed significant teratogenic or adverse pregnancy risks, underscoring the substantial heterogeneity in exposure assessment methods, study design, and confounder control [5,23]. Reviews of nonionizing radiation effects have also noted that while EMW exposure may induce cellular stress responses, such as oxidative stress or altered signaling pathways, the direct clinical relevance of these mechanisms to pregnancy outcomes remains insufficiently established, reinforcing the need for rigorous and balanced systematic synthesis [24].

### Strengths and Limitations

These inconsistencies highlight the need for a comprehensive and methodologically rigorous systematic review. Many existing studies rely on self-reported device use or indirect exposure proxies, which may introduce recall bias and exposure misclassification [25]. Additionally, limited adjustment for confounding factors such as maternal stress, occupational radiation exposure, environmental pollutants, and thermal stress further complicates the interpretation of the available evidence [26].

The strengths of this protocol include adherence to PRISMA-P guidelines, a comprehensive search strategy, and the use of contemporary, domain-based risk of bias assessment tools appropriate for observational evidence, with explicit consideration of confounding and exposure misclassification. Nonetheless, heterogeneity in exposure metrics, outcome definitions, and study populations may limit the certainty of any conclusions that can be drawn; therefore, the findings will be interpreted with appropriate caution.

### Implications and Future Directions

By synthesizing evidence from human observational studies, this systematic review aims to improve understanding of potential associations between nonionizing EMW exposure and pregnancy outcomes. Evidence from experimental and modeling studies will be discussed contextually in the Discussion to support biological plausibility, without being included in the quantitative synthesis. Experimental studies have suggested that RF exposure may influence oxidative balance, mitochondrial integrity, and calcium signaling in placental and fetal tissues under specific experimental conditions [20,27].

The anticipated contributions of this review are threefold: (1) to synthesize fragmented evidence regarding possible associations between EMW exposure and pregnancy outcomes while carefully accounting for study quality and bias; (2) to critically evaluate exposure assessment methodologies and identify research gaps, particularly regarding real-time dosimetry and gestational timing of exposure; and (3) to support evidence-informed discussion and future research directions rather than definitive risk conclusions, consistent with precautionary principles proposed by international organizations such as the WHO and ICNIRP.

### Dissemination Plan

The findings of this systematic review will be disseminated through publications in peer-reviewed open-access journals and presentations at relevant scientific conferences. In addition, the results may be communicated to academic and public health stakeholders to facilitate transparent, evidence-based interpretation and inform future research priorities.

### Conclusion

This protocol provides a structured and reproducible framework for systematically synthesizing evidence of nonionizing EMW exposure during pregnancy. By applying rigorous methodological standards and transparent quality assessments, the forthcoming review aims to clarify the current state of evidence while explicitly acknowledging its limitations. The conclusions will be framed cautiously, reflecting the strength and consistency of the available data and are intended to support responsible scientific interpretation and future research rather than draw definitive clinical or causal inferences.

## Supplementary material

10.2196/86479 Checklist 1 PRISMA 2020 checklist.

## Abbreviations

EMF: electromagnetic field
EMW: electromagnetic wave
GRADE: Grading of Recommendations Assessment, Development, and Evaluation
PICOS: Population Intervention (or Exposure, for observational studies) Comparator Outcomes Study design
PRISMA-P: Preferred Reporting Items for Systematic Review and Meta-Analysis Protocols
PROSPERO: International Prospective Register of Systematic Reviews
RF: radiofrequency
STROBE: Strengthening the Reporting of Observational Studies in Epidemiology
